# Ferroptosis: new insight into the mechanisms of diabetic nephropathy and retinopathy

**DOI:** 10.3389/fendo.2023.1215292

**Published:** 2023-08-03

**Authors:** Luxin Li, Yucen Dai, Dan Ke, Jieting Liu, Peijian Chen, Dong Wei, Tongtong Wang, Yanjie Teng, Xiaohuan Yuan, Zhen Zhang

**Affiliations:** ^1^ College of Life Sciences, Mudanjiang Medical University, Mudanjiang, China; ^2^ Heilongjiang Key Laboratory of Anti-Fibrosis Biotherapy, Mudanjiang Medical University, Mudanjiang, China; ^3^ Department of Ophthalmology, Affiliated Hongqi Hospital, Mudanjiang Medical University, Mudanjiang, China; ^4^ Department of Endocrinology, Affiliated Hongqi Hospital, Mudanjiang Medical University, Mudanjiang, China; ^5^ School of First Clinical Medical College, Mudanjiang Medical University, Mudanjiang, China

**Keywords:** diabetic nephropathy, diabetic retinopathy, ferroptosis, regulatory cell death, oxidative stress

## Abstract

Diabetic nephropathy (DN) and diabetic retinopathy (DR) are the most serious and common diabetes-associated complications. DN and DR are all highly prevalent and dangerous global diseases, but the underlying mechanism remains to be elucidated. Ferroptosis, a relatively recently described type of cell death, has been confirmed to be involved in the occurrence and development of various diabetic complications. The disturbance of cellular iron metabolism directly triggers ferroptosis, and abnormal iron metabolism is closely related to diabetes. However, the molecular mechanism underlying the role of ferroptosis in DN and DR is still unclear, and needs further study. In this review article, we summarize and evaluate the mechanism of ferroptosis and its role and progress in DN and DR, it provides new ideas for the diagnosis and treatment of DN and DR.

## 1 Introduction

Diabetes is the most common metabolic disorder in the world, and its incidence rate has risen sharply. The International Diabetes Federation estimates that 537 million people worldwide had diabetesin 2021, a number that is expected to rise to 783 million by 2045 (1). Diabetic vascular complications are one of the main causes of death and disability (2). The microvascular damage due to hyperglycemia can lead to diabetic microvascular complications, including diabetic nephropathy (DN) and diabetic retinopathy (DR). DN is the leading cause of chronic kidney failure and end-stage kidney disease. Oxidative stress induced by chronic hyperglycemia is considered a key mechanistic factor in the development of DN. DN is characterized by persistent albuminuria, elevated arterial blood pressure, and decreased glomerular filtration rate (3). Renal function in patients with diabetes frequently worsens in severity over time, and eventually to renal failure. The pathological changes are similar since both DN and DR have microvascular changes. Hyperglycaemia induces retinal oxidative stress, which plays an important role in DR (4). DR is a leading cause of progressive vision loss and blindness (5). The current research hotspots mainly focus on the exploration of the pathological mechanism of DN and DR.

Ferroptosis, an iron-dependent lipid peroxidation-driven new form of regulated cell death, is different from other types of cell death such as apoptosis, necrosis, autophagy, and pyroptosis (**Table 1**) (6, 7). Iron deposition leads to the formation of reactive oxygen species (ROS) and oxidative stress through the Fenton reaction, further promoting lipid peroxidation, a key factor in ferroptosis (8). Emerging evidence suggests a strong link between diabetic complications and ferroptosis. Hyperglycemia leads to the overproduction of ROS and induces oxidative stress in various organs of diabetic patients (9). High glucose could cause an overload of iron, and iron dysregulation induces the production of ROS and promotes oxidative stress, which leads to ferroptosis finally (10). Studies have shown that high ROS induces oxidative stress in kidney and retinal cells resulting in cell death (11, 12). This means that targeting ferroptosis may be an effective strategy to treat related DN and DR. This review summarized the mechanisms of ferroptosis and discussed the role of ferroptosis in DN and DR. In addition, we elaborated recent data about the novel actors of some “conventional” drugs or natural compounds as ferroptosis inducers or inhibitors to find common molecular mechanisms and therapeutic targets in DN and DR. It is of great significance to clarify the pathological mechanism of diabetic microvascular complications.

**Table 1:** Comparison of several common types of cell death.

Cell death	Ferroptosis	Apoptosis	Autophagy	Necroptosis	Pyroptosis
Inducing factor	Accumulation of iron ions.	Gene regulation under normal physiological regulation.	Nutrient deficiency or hormonal induction.	Pathological factors or injuries are passively triggered.	Inflammation-induced activation of promoter and proteolytic activation of GSDMD.
Morphological features	Small mitochondria, cristae reduction, membrane density increase, mitochondrial membrane rupture, but normal nucleus.	Nuclear rupture, plasma membrane blebbing, cell shrinkage, formation of apoptotic bodies, and phagocytosis of neighboring cells.	Accumulation of autophagic vacuoles, vacuolization of the cytoplasm, and absence of chromatin condensation.	Loss of plasma membrane integrity and release of cytoplasmic contents, swelling of cytoplasm and organelles, and condensation of chromosomes.	Plasma membrane disruption and release of cellular contents and proinflammatory cytokines.
Detection mode	Cell viability assay: CCK-8, intracellular iron level, reactive oxygen species (ROS) level, and changes in death-related factors such as COX-2, ACSL4, PTGS2, NOX1, GPX4 and FTH1.	Mitochondrial membrane potential detection, Annexin V/PI, TUNEL assay, apoptosis-related pathways, and apoptosis-related proteins.	The levels of autophagy-related proteins such as Atg5, Atg7, BeclinI, LC3, and P62 were detected, and autophagosome fluorescent single/double labeling method and lysosomal function were detected.	Transmission electron microscope or scanning electron microscope observation, Annexin V/PI.	Gasdermin D, Caspase-1, Caspase-4 IL-1β, IL-18 and other indicators

## 2 Mechanisms of ferroptosis

**Figure 1:**
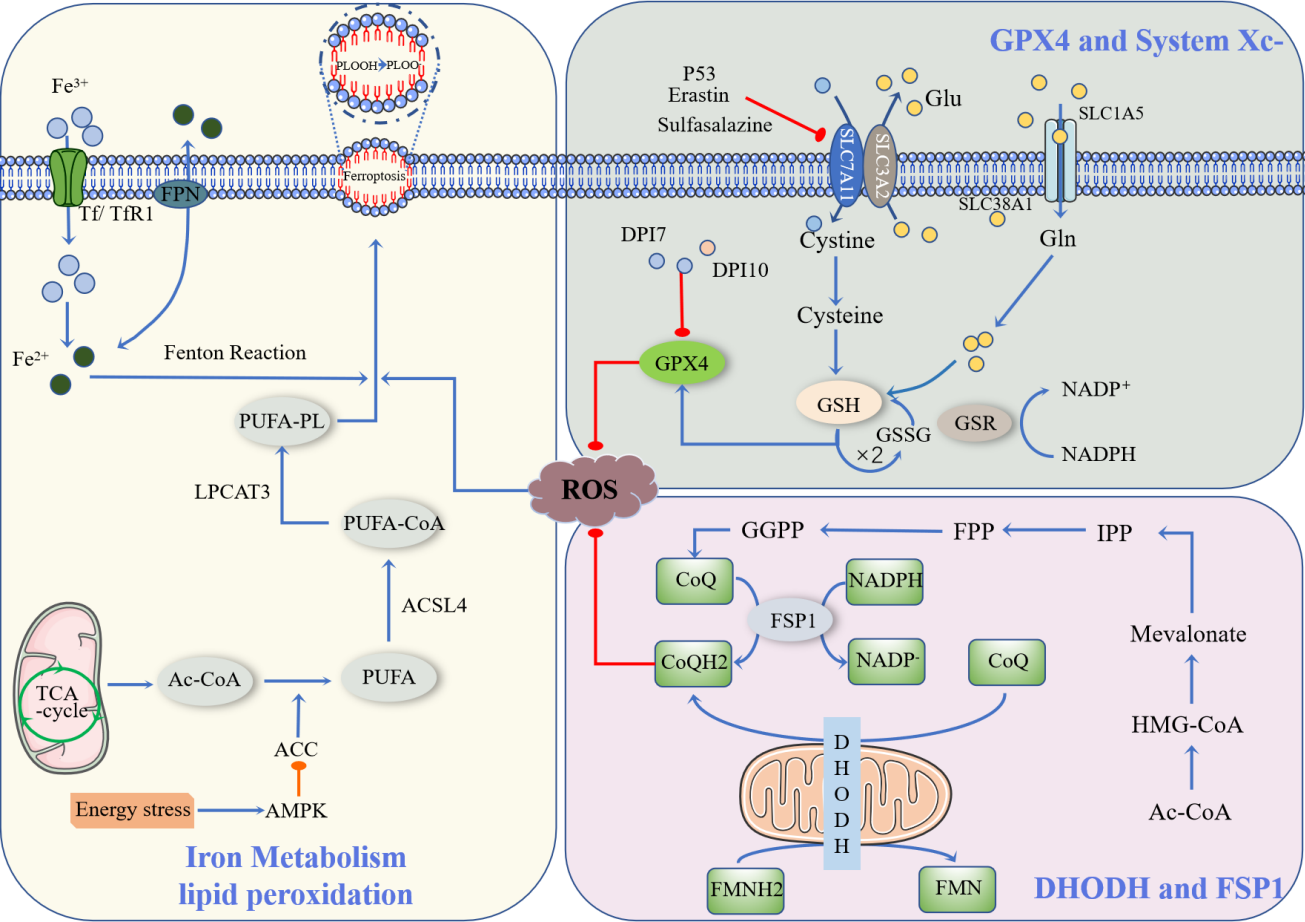
Occurrence and protective factors of Ferroptosis. Cystine and Glu are retroactively transported via the SLC7A11/SLC3A2 complex (p53, Erastin, Sulfasalazine, etc., can inhibit the complex). The cystine that enters the cell is transformed into cysteine by GSH biosynthesis using the electrons supplied by NADPH/H^+^, and oxidized glutathione (GSSG) is reduced by glutathione disulfide reductase (GSR). They finally enhance the activity of glutathione peroxidase 4 (GPX4). DHODH and FSP1 inhibit the occurrence of ferroptosis through CoQ/CoQH2, and Ac-CoA generates CoQ through a series of biosynthesis reactions. NADPH, as a hydrogen donor, assists FSP1 in reducing CoQ to CoQH2, while DHODH performs this process via FMNH2. CoQH2, in a reduced state, inhibits ferroptosis. Ac-CoA in mitochondria is catalyzed by ACC to produce PUFA, and PUFA is further catalyzed by ACSL4 to form PUFA-CoA, which is then activated by LPCAT3 as PUFA-PL, affecting the transmembrane properties of polyunsaturated fatty acids and thus promoting ferroptosis. Ferric ions are obtained by cells mainly through Tf/TfR1 protein transport and are acidified by endocytic spheroids and released from FPN into the cytoplasm as ferric ions. Excess iron further produces ROS through the Fenton reaction, resulting in an iron drop.

Ferroptosis is a newly described type of iron-dependent programmed cell death. The basic principle is that divalent iron or ester oxygenase catalyzes lipid peroxidation of highly expressed unsaturated fatty acids on the cell membrane, which induces cell death (13). In addition to increased levels of peroxidation, it also reduces the decrease of glutathione peroxidase 4 (GPX4), the core enzyme regulating the antioxidant system (glutathione system, GSH system) (14). Ferroptosis is regulated by multiple signaling pathways (**Figure 1**) by the following mechanisms.

### 2.1 GPX4-dependent ferroptosis defense pathway

Currently, the known upstream pathways of ferroptosis ultimately point directly or indirectly to glutathione peroxides (GPXs), and therefore, affect the activity of GPXs by regulating GPX4, which is one of the most important ferroptosis defense pathways available (15). Among them, the GSH/GPX4 pathway is the main regulatory mechanism of ferroptosis, such as in the regulation of GPX4, inhibition of the cystine/glutamate reverse transporter system (System Xc^-^), glutamine metabolic pathway, and p53 regulatory axis (16).

GPX4 is an inhibitor of lipid peroxidation that degrades small molecule peroxides and relatively complex lipid peroxides (17). There are many members of the GPXs, including GPX1-GPX8, of which GPX4 plays an important role in ferroptosis. Yang et al. found that a decrease in GSH led to a decrease in GPX activity (18). GPX catalyzes hydrogen peroxide and degradation of hydrogen peroxide and inhibits the production of lipid reactive oxygen species, for which glutathione is an essential cofactor. The classical ferroptosis control axis requires cystine reduction to cysteine, GSH and/or thioredoxin reductase 1(TXNRD1)-dependent reduction of cystine to cysteine, GSH biosynthesis, and GPX4-mediated reduction of phospholipid hydroperoxide (PL-OOH) to its corresponding alcohol phospholipid hydroxide (PL-OH) using electrons provided by NADPH/H^+^, oxidized glutathione (GSSG) is recovered by glutathione disulfide reductase (GSR), GSH synthesis is inseparable from glutamine (Gln) and intracellular glutamine uptake is mainly dependent on the involvement of SLC1A5/SLC38A1 (19).

System Xc^-^ is a heterodimer composed of SLC7A11 and SLC3A2, of which SLC7A11 is the main subunit that exerts its function (20). Downregulation of SLC7A11 can indirectly inhibit GPX4 activity by inhibiting the cysteine metabolic pathway, leading to reduced intracellular cystine levels and depletion of GSH biosynthesis, which in turn leads to lipid peroxide accumulation and ultimately induces cell ferroptosis (21). In addition, Erastin, a classical ferroptosis promoter, can target and inhibit SLC7A11 to induce ferroptosis (22). Related studies have shown that the ferroptosis process induced by sulfasalazine is also associated with inhibition of SLC7A11 (23). In addition, SLC7A11 is also associated with the p53 gene: p53 downregulates SLC7A11 expression and inhibits Systemic Xc^-^ uptake of cysteine, leading to decreased cysteine-dependent glutathione peroxidase activity, reduced cellular antioxidant capacity, and increased lipid reactive oxygen species, resulting in cellular iron sagging (16). Inhibition of Systemic Xc^-^ would lead to compensatory transcriptional upregulation of SLC7A11, preventing cystine uptake and thus leading to ferroptosis (24).

Cystine is an indispensable component necessary for the biosynthesis of GSH, a process catalyzed by glutamate-cysteine ligase and glutathione synthase, and a decrease in cellular antioxidant capacity (25). Glutathione is an essential cofactor for the function of GPXs, which results in a decrease in GPX activity. During glutamate-induced excitotoxic cell death, glutamate can initiate two pathways, one for calcium influx and the other for inhibition of Systemic Xc^–^dependent cysteine uptake pathway (24). Calcium chelators have no inhibitory effect on Erastin-induced ferroptosis, whereas excessive glutamate levels impede the function of Systemic Xc^-^ and induce ferroptosis. Reduction of Systemic Xc^-^ activity decreases cystine uptake; therefore, reduced cystine uptake leads to reduced GSH and eventually GPX4 activity, decreased cellular resistance to peroxidation, accumulation of lipid reactive oxygen species, and oxidative cell death (26).

p53 is an important tumor suppressor gene that mediates cell cycle inhibition, senescence, and apoptosis and plays an important role in tumorigenesis and progression (27). Recently, it has been shown that activated p53 can bind to the promoter region of the SLC7A11 gene, which inhibits the transcriptional activity of the SLC7A11 gene and affects the synthesis of GSH, which can induce the occurrence of ferroptosis (28). Jiang et al. also found that the messenger RNA and protein expression of SLC7A11 decreased significantly after upregulation of p53 gene expression, which confirmed that SLC7A11 is a new target of the p53 gene (29). p53 inhibits Systemic Xc^-^ uptake of cystine by downregulating the expression of System Xc^–^component SLC7A11, leading to a decrease in cystine-dependent glutathione peroxidase activity, a decrease in cellular antioxidant capacity, and an increase in lipid reactive oxygen species, resulting in cellular ferroptosis (16).

In addition to the above-mentioned indirect action of acting on GSH that activates the GPX4 enzyme, it is also possible to eliminate GPX4 directly, such as Diphenyleneiodonium chloride-7(DPI-7), Diphenyleneiodonium chloride-10 (DPI-10), GPX4 inhibitors, squalene synthase, HMG-CoA reductase, etc (30). In summary, whether by direct or indirect means, the ultimate goal is to regulate the core enzyme GPX4, which can inhibit ferroptosis by eliminating lipid peroxides using reduced GSH.

### 2.2 GPX4-independent ferroptosis defense pathway

Recent studies have identified ferroptosis suppressor protein 1 (FSP1) and the mitochondrial enzyme dihydroorotate dehydrogenase (DHODH) as GPX4-independent ferroptosis defense pathways, both of which inhibit ferroptosis by using the reduced state of coenzyme Q10 (CoQ10) to prevent cell membrane lipid peroxidation and thus inhibit the occurrence of ferroptosis (31).

FSP1, formerly also known as apoptosis-inducing factor mitochondria-associated (AIFM2), was renamed FSP1 to avoid confusion due to the recent discovery of its role in inhibiting cellular ferroptosis (32). The inhibition of ferroptosis by FSP1 is mediated by CoQ10, which reduces ubiquinone (CoQ) to dihydro ubiquinone (CoQH2) in the cell membrane, and CoQH2 acts as an antioxidant blocking lipid peroxidation inhibiting ferroptosis from occurring (33).

Mitochondria are organelles wrapped by two membranes, the inner and outer membrane, and are the main site of aerobic respiration, with large amounts of ROS generated by the electron transfer process in the inner membrane (34). In addition to the synthesis of pyrimidine nucleotides, DHODH was found to inhibit ferroptosis in mitochondria by generating CoQH2 in the inner mitochondrial membrane, because CoQH2 can act as a radical trapping antioxidant to prevent lipid peroxidation and thus inhibit ferroptosis (35).

### 2.3 Iron metabolism and lipid peroxidation

Acetyl coactivators in mitochondria produce the polyunsaturated fatty acids (PUFA) catalyzed by acetyl-CoA carboxylase (ACC) (36). However, the presence of PUFA does not directly lead to ferroptosis. This requires further catalysis by members of the long-chain acyl-coenzyme A synthases (ACSL) family, especially ACSL4, to form PUFA-CoA, which is then activated by LPCAT3 to PUFA-PL, affecting the transmembrane properties of polyunsaturated fatty acids and thus promoting ferroptosis (37). Activation of AMP-activated protein kinas (AMPK) by energy stress directly inhibits ACC, which limits PUFA synthesis and thus inhibits iron phagocytosis (38).

Cellular uptake of iron is mainly through the Tf/TfR1 protein transport pathway to obtain trivalent iron ions, but these trivalent irons still need to be released from FPN as divalent iron by acidification of endocytic cells (39). The divalent iron then enters the cytoplasm through the endosomes. Excess iron further generates ROS through the Fenton reaction, which leads to ferroptosis (40).

## 3 Ferroptosis and diabetic microangiopathy

### 3.1 Ferroptosis and diabetic nephropathy

DN is the most common complication of diabetes mellitus and the leading cause of end-stage renal disease, which is a “global medical disaster” because of the difficulty of diagnosis and management (41, 42). However, the pathogenesis and pathophysiology of DN are complex. Therefore, further studies on its molecular mechanisms are needed to develop new therapeutic approaches.

Ferroptosis is a newly discovered form of regulated cell death that is different from apoptosis, necrosis, autophagy, and other forms and depends on iron and ROS. It is regulated by a variety of cellular metabolic pathways and various signaling pathways related to diseases (19). Ferroptosis can produce a large amount of ROS, and the kidney is sensitive to oxidative stress due to its rich mitochondrial structure, suggesting that ferroptosis may be related to DN. The persistent hyperglycemic environment promotes ROS production and increases basal levels of several renal cell types, including mesangial cells, podocytes, and tubular epithelial cells (43). The identification of related genes also reveals the involvement of ferroptosis in the pathogenesis of DN (44). Recently, it was shown that inhibition of ferroptosis may slow the progression of DN in a variety of cellular and animal models.

Mesangial cells are a special type of smooth muscle cells distributed between the capillary rings of glomerular capillaries, and their damage is the basic pathological change of DN (45). Sustained hyperglycemia induces hyperplasia of the glomerular mesangium, leading to thickening of the basement membrane, sclerosis of the glomerular capillary wall, and ultimately proteinuria (46). High mobility group box 1 (HMGB1) is known to act as a pro-inflammatory cytokine that is involved in many diabetic complications (47). Related experiments have shown that high glucose (HG)-induced ferroptosis of mesangial cells can be prevented by inhibiting HMGB1 (48). Ferroptosis is also associated with podocyte injury in diabetic patients. Podocytes are an important component of the glomerular filtration barrier (GFB), and recently, it was revealed that podocytes regulate the GFB through endocytosis, and podocyte injury is considered one of the main mechanisms leading to GFB damage (49, 50). GPX4 and SLC7A11 are considered key proteins in the prevention of ferroptosis, and the loss or inhibition of GPX4/SLC7A11 can induce ferroptosis (51). *In vitro* studies demonstrate that Sp1-mediated upregulation of Prdx6 expression inhibits iron accumulation and increases the expression of SLC7A11 and GPX4, preventing podocyte injury in DN (52). Another study also revealed that Ginkgolide B may improve DN by protecting the kidney from ferroptosis and oxidative stress damage by inhibiting the ubiquitination of GPX4 (53).

Renal tubular damage is the key factor of DN. The formation of advanced glycation end products (AGEs) associated with hyperglycemia plays a central role in the pathogenesis of diabetic nephropathy. High glucose induces oxidative stress by increasing the production of ROS, excessive iron in cells damages cell function by producing ROS, and renal tubular cells are more sensitive to oxidative stress and lipid peroxidation (54, 55). Wang et al. (56) establish a DN model using streptozotocin (STZ) and db/db mice and found that the expression levels of ferroptosis-related markers, ACSL4 and GPX4, were increased in kidney tissue, especially in the renal tubules. Rosiglitazone, an ACSL4 inhibitor, has a protective effect on DN mice by attenuating ferroptosis, which provides a possibility for the treatment of DN with ferroptosis. In addition, some drugs for diabetes, such as dapagliflozin, improve renal tubular injury by inhibiting ferroptosis (51). Recently, some researchers have found that empagliflozin prevents the progression of ferroptosis by promoting AMPK-mediated (the transcription factor nuclear factor red line related factor 2) Nrf2 activation pathway *in vivo* and vitro (57). Chinese medicine’s active ingredients, such as licorice flavone, calycosin, and 7-hydroxycoumarin, also inhibit ferroptosis and reduce renal tubular damage induced by diabetes to achieve the effect of DN treatment (58–60). These results suggest that ferroptosis mediates renal tubular injury in DN, and inhibiting ferroptosis in renal tubular cells may play a role in the treatment of DN.

Recent studies have shown that ferroptosis is involved in renal fibrosis of DN, which is the terminal pathological change of DN (56). Heme oxygenase-1 (HO-1) plays an important role in anti-oxidative stress injury, and hypoxia-inducible factor-1 (HIF-1) is a key molecule involved in alleviating hypoxia-induced injury. Excessive HO-1 degradation of oxidized heme leads to iron overload, which results in oxidative stress and lipid peroxidation (61). Feng et al. (62) demonstrated that diabetes enhanced renal tubular damage accelerated tubular iron overload, and aggravated the accumulation of ROS in mouse kidneys through the HIF-1α/HO-1 pathway. In contrast, the ferroptosis inhibitor, Ferrostatin-1, inhibited renal tubular iron overload, renal oxidative stress, and lipid peroxidation in diabetic mice. Renal tubular damage in diabetes may be considered a major cause of renal fibrosis. A large number of studies have shown that the TGF-β/Smad signaling pathway plays a crucial role in renal fibrosis (63). Transforming growth factor-β1 (TGF-β1) is an important factor leading to renal fibrosis, and relevant studies have reported that TGF-β1-induced renal tubular cell death is related to DN (64). Nrf2 is an important regulator of the antioxidant system and realizes redox reactions with multiple downstream targets. Salusin-β participates in HG-induced ferroptosis in HK-2 cells in an Nrf2-dependent manner (65). Fenofibrate inhibits ferroptosis and delays the progression of diabetic nephropathy by up-regulating Nrf2 and 7-hydroxycoumarin by activating the Nrf-2/HO-1 pathway (60, 66). Notoginsenoside R1 (NGR1) is a novel saponin derived from Panax notoginseng. NGR1 promotes Nrf2-mediated HO-1 expression to prevent DN, eliminates ROS that induces apoptosis and TGF-β signaling, and plays a renoprotective role in DN by inhibiting oxidative stress-induced apoptosis and renal fibrosis (67).

In addition, several researchers reviewed the latest findings and emerging trends in ferroptosis research and highlighted that the tumor suppressor p53 has a dual role in ferroptosis. On the one hand, p53 enhances ferroptosis by inhibiting SLC7A11 expression or promoting SAT1 and GLS2 expression. On the other hand, p53 inhibits ferroptosis by inhibiting DPP4 activity or inducing CDKN1A/p21 expression (68). Our previous studies also confirmed that hyperglycemia regulates pathogenic processes in DN through a miR-23b/G3BP2 feedback circuit involving p38MAPK and p53 (69). This suggests that p53 may be an attractive therapeutic target for regulating ferroptosis against DN. Some researchers have analyzed the mechanisms, pathways, and genes related to ferroptosis in DN through bioinformatics, revealing Hub genes related to ferroptosis in DN that mainly include FPR3, C3AR1, CD14, ITGB2, RAC2, and ITGAM. Moreover, there are non-coding genes that interact with the Hub genes and these mainly include has-miR-572, has-miR-29a-3p, has-miR-29b-3p, has-miR-208a-3p, has-miR-153-3p, has-miR-29c-3p, etc. The transcription factors related to DN mainly include HIF1α, KLF4, KLF5, RUNX1, SP1, VDR, and WT1. A KEGG pathway enrichment analysis showed that the MAPK signaling pathway was significantly enriched (70–72). These observations also serve as a starting point for future mechanistic studies. In conclusion, ferroptosis plays a crucial role in the development of DN, and ferroptosis may be the future direction of DN treatment.

### 3.2 Ferroptosis and diabetic retinopathy

DR is a common and specific microvascular complication of diabetes mellitus. Blindness caused by DR has a great impact on the life of patients (73). The disease is negatively affected by diabetes mellitus, which alters normal cellular interactions, leading to severe vascular abnormalities, the loss of the blood-retinal barrier, and impaired neuronal function. Conventional therapies have not responded well to recent treatments, and a new treatment is needed (74). With evidence supporting the interaction between iron metabolism and diabetes, the molecular mechanism of ferroptosis in the pathogenesis of DR has also attracted attention, which provides a new therapeutic target for the treatment of DR (75).

Retinal pericyte loss is one of the earliest changes associated with DR. Although the pathophysiological mechanisms of DR are complex, vascular endothelial damage, increased vascular permeability, and neovascularization are the most common phenomena (76). Vascular endothelial growth factor (VEGF) plays a leading role in the occurrence and progression of DR, and berberine inhibits insulin-induced retinal endothelial cell activation through the Akt/mTOR/HIF-1α/VEGF pathway to improve insulin-induced DR progression (77). Increased vascular permeability in the early stage of DR leads to macular edema in the later stage, which is related to the release of proinflammatory cytokines (74). TRIM46 promotes HG-induced ferroptosis in human retinal capillary endothelial cells (HRCECs) by regulating the ubiquitination and degradation of GPX4 (76). A recent study showed that TRIM46 aggravates HG-induced hyperpermeability and inflammatory response of HRCECs by promoting IκBα ubiquitination (78). In addition, ferroptosis is characterized by the accumulation of lipid ROS, particularly the loss of the activity of lipid hydroperoxides and the lipid repair enzyme GPX4 (54). Fan et al. (79) found that BMS309403, an inhibitor of FABP4, promoted peroxisome proliferator-activated receptor γ (PPARγ), thereby regulating PPARγ-mediated ferroptosis to alleviate lipid peroxidation and oxidative stress in DR.

Studies on non-coding RNAs and related signaling pathways demonstrate a link between DR and ferroptosis. Previous research by our group showed that miR-200b was closely associated with diabetes and its complications (80–82), and miR-200b regulated VEGF-mediated alterations in DR (80). Zhu et al. (83) found that the knockdown of circ-PSEN1 reduced ferroptosis in ARPE19 cells induced by high glucose through the miR-200b-3p/CFL2 axis. Zhou et al. (84) also found that HG-induced ferroptosis in retinal epithelial cells could be inhibited by blocking the miR-338-3p/SLC1A5 axis. In addition, Astragaloside-IV may inhibit DR by reducing miR-138-5p expression and subsequently increasing Sirt1/Nrf2 activity and cellular antioxidant capacity to alleviate ferroptosis, resulting in reduced cell death (85). In addition, Liu et al. (86) expanded the understanding of the relationship between autophagy and ferroptosis. They found that the GMFB antibody, lysosomal activator NKH477, CMA activator QX77, and ferroptosis inhibitor lipstatin-1 effectively prevented early DR, which had a strong clinical application value. In summary, ferroptosis plays a crucial role in the development of DR and may provide a new therapeutic strategy for the treatment of DR.

## 4 Conclusion and perspectives

This article reviews the role and potential mechanisms of ferroptosis in DN and DR. Although diabetes has been investigated for decades, the effective prevention and treatment of diabetic complications is a challenging clinical issue. Long-term hyperglycemia and genetic susceptibility increase the risk of microvascular complications in patients with diabetes (87, 88). The microvascular complications of diabetes are related to the long-term damage and dysfunction of various organs and systems, including the kidney and retina, which may lead to end-stage renal disease and blindness, leading to a significant increase in incidence rate and mortality (89, 90). DN and DR are interrelated through a common pathophysiological mechanism. The main biological mechanism of DN and DR can be linked through the excessive production of ROS, downstream intracellular signal pathways their regulators, which can be used as therapeutic targets for the treatment of diabetes complications (91, 92).

**Figure 2:**
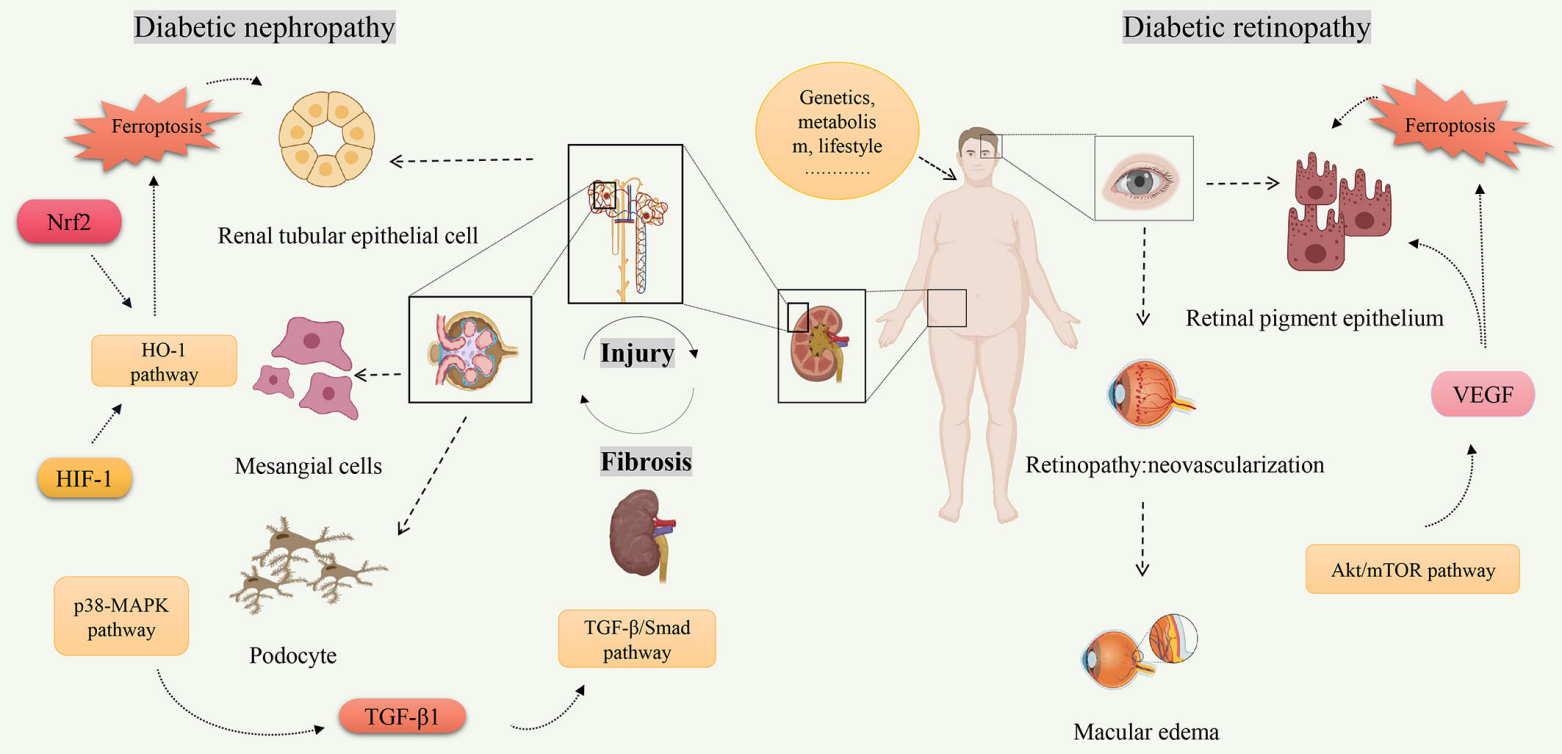
The related signaling pathways on ferroptosis of diabetic nephropathy and diabetic retinopathy. Created by Biorender.com. High-risk factors for diabetes (increasing age, overweight or obesity, etc.) are closely related to the occurrence of diabetes. Hyperglycemia is one of the main clinical manifestations of diabetes, and also one of the high-risk factors leading to diabetes complications. Molecules (Nrf2, HIF-1, TGF-β1, VEGF, etc.) regulate ferroptosis through multiple signaling pathways (TGF-β/Smad pathway, Akt/mTOR pathway, HO-1 pathway, etc.) and play a role in pathological progression of diabetic nephropathy and diabetic retinopathy.

Ferroptosis is a new form of cell death characterized by iron-dependent accumulation of lipid peroxides to lethal levels (93). Iron is an essential trace metal element involved in many physiological processes of the human body. However, excess iron can generate oxidative stress and cause tissue damage (94). Given the association between diabetes and ferroptosis, starting from the key target of ferroptosis may improve diabetes. In recent years, there has been increasing research on ferroptosis in DN and DR. The pathophysiology of DN and DR is complex, which involves many types of cells. Multiple signaling pathways are involved in the ferroptosis process of DN and DR, including Nrf2, HIF-1, TGF-β1, VEGF, etc (**Figure 2**). Some ferroptosis inhibitors and iron chelators have shown good regulatory effects in animal and cellular experiments related to DN and DR. Many antiferroptotic natural products and drugs also provide new ideas and targets for the treatment of DN and DR (**Table 2**). Although selective inhibition of ferroptosis has been proven to substantially improve kidney function and play a retinal protected role in various animal models and cell models, clinical trials have not yet been performed with ferroptosis-specific inhibitors to treat DN and DR. The exact mechanism of ferroptosis needs to be explored through further study. Monitoring and controlling ferroptosis related factors may be a promising measure for early diagnosis and treatment of diabetes. However, there have been no established specific markers to demonstrate the presence of ferroptosis *in vivo*. Identifying key biomarkers of ferroptosis will also facilitate our understanding of its role and progress in diabetic complications. To sum up, although its biological function and molecular mechanism have not been thoroughly elucidated, ferroptosis has become a hot spot in diabetic complications research.

**Table 2:** Drugs or targets against ferroptosis in diabetic nephropathy and retinopathy.

Disease	Compound/target	Cell type/animal model	Effect	Mechanism	Ref.
Diabetic nephropathy	HMGB1	SV40-MES 13 cells	Inhibition	HMGB1 regulates ferroptosis through the Nrf2 pathway by preventing ROS and LDH generation, decreasing ACSL4, PTGS2, and NOX1, and increasing GPX4 levels in mesangial cells in mesangial cells.	(48)
	Prdx6	MPC5 cells	Inhibition	Sp1-mediated upregulation of Prdx6 expression decreases HG-induced ferroptosis by increasing ACSL4 expression levels in podocytes.	(52)
	Rosiglitazone	STZ &db/db mice	Inhibition	Rosiglitazone decreases the expression levels of ACSL4 and increases the expression levels of GPX4 to alleviate ferroptosis and reduce IL-6, TNF-α, and Ptgs2.	(56)
	Dapagliflozin	HK-2 cells/HFD&STZ-induced C57BL/6 mice	Inhibition	Dapagliflozin ameliorates ferroptosis in HK-2 cells by SLC40A1 stabilization.	(51)
	Empagliflozin	Erastin or HG-induced HK-2 cells/T2DM mice	Inhibition	Empagliflozin attenuates renal tubular ferroptosis through the AMPK/NRF2 pathway.	(57)
	Calycosin	HK-2 cells/db/db mice	Inhibition	Calycosin relieves the induction of ferroptosis by reducing lipid ROS, LDH, and NCOA4 levels.	(17)
	Umbelliferone	HK-2 cells/C57BLKS/J db/db mice	Inhibition	Umbelliferone attenuates the level of high glucose-induced ferroptosis by activating the Nrf2/HO-1 pathway.	(60)
	Salusin-β	HK-2 cells	Induction	Salusin-β serves as an inducer of oxidative stress in HK-2 cells by increasing ROS levels and decreasing GSH activities.	(65)
	Fenofibrate	HK-2 cells/STZ-induced DBA/2J diabetic mice	Inhibition	Fenofibrate up-regulates Nrf2 to inhibit diabetes-related ferroptosis.	(66)
	P53	HCT116/SW48 cells/Tumour-bearing mice	Induction	P53 enhances ferroptosis by inhibiting the expression of SLC7A11, SAT1, and GLS2.	(68)
			Inhibition	P53 suppresses ferroptosis through the direct inhibition of DPP4 activity or by the induction of CDKN1A/p21 expression.	
Diabetic retinopathy	TRIM46	HRCECs	Induction	TRIM46 facilitates GPX4 ubiquitination and contributes to high glucose-induced ferroptosis and cell growth inhibition in human retinal capillary endothelial cells.	(76)
	FABP4	ARPE-19 cells/STZ into C57BL/6 male mice	Induction	Inhibiting FBP4 alleviates lipid peroxidation and oxidative stress in DR by regulating PPARγ-mediated ferroptosis.	(79)
	Circ-PSEN1	ARPE19 cells	Induction	Knockdown of circ-PSEN1 mitigates ferroptosis of ARPE19 cells induced by HG via the miR-200b-3p/CFL2 axis.	(83)
	MiR-338-3p/SLC1A5 axis reprograms retinal pigment epithelium	RPE cells	Inhibition	MiR-338-3p/SLC1A5 axis blocks high glucose-induced ferroptosis in RPE cells.	(84)
	Astragaloside-IV	RPE cells	Inhibition	AS-IV inhibits the miR-138-5p expression and increases Sirt1/Nrf2 activity to alleviate ferroptosis.	(85)
	Glia maturation factor-β	RPE cells	Induction	Glia maturation factor-β induces ferroptosis by impairing chaperone-mediated autophagic degradation of ACSL4 in early diabetic retinopathy.	(86)

## Author contributions

Conceptualization, LL and ZZ; original draft preparation, LL, YD and DK; collected the literatures, YD, DW and TW; review and editing, XY and ZZ; supervision, YT, JL, and PC. All authors contributed to the article and approved the submitted version.
